# A Rare Case of Juvenile Psoriatic Arthritis

**DOI:** 10.7759/cureus.72985

**Published:** 2024-11-04

**Authors:** Alexandru Dinulescu, Ana Prejmereanu, Daniela Pacurar, Oana Neagu, Irina Dijmarescu

**Affiliations:** 1 Pediatrics, Carol Davila University of Medicine and Pharmacy, Bucharest, ROU; 2 Pediatrics, Emergency Hospital for Children "Grigore Alexandrescu", Bucharest, ROU; 3 Pathology, Carol Davila University of Medicine and Pharmacy, Bucharest, ROU; 4 Pathology, Emergency Hospital for Children "Grigore Alexandrescu", Bucharest, ROU

**Keywords:** juvenile arthritis, juvenile idiopathic arthritis (jia), juvenile psoriatic arthritis (jpsa), pediatric psoriasis, psoriasis

## Abstract

This is a case report of a one-year-and-nine-month-old girl with multiple guttate psoriasis skin lesions, non-traumatic knee arthritis, and no family history of autoimmune diseases. Laboratory tests revealed no suggestive markers of juvenile psoriatic arthritis (JPsA), while the dermatological examination described scaly erythematous lesions with a positive Auspitz sign. The diagnosis was confirmed by a skin biopsy and histopathological examination. The psoriasis lesions were preceded by the onset of arthritis within two weeks. The negative family history, age of onset, clinical presentation, and negative antinuclear antibodies make it a particular case of JPsA.

## Introduction

Psoriasis is a chronic, inflammatory, immune-mediated disorder that is clinically heterogeneous; it is a systemic disease that primarily affects the skin, with the most important and common extracutaneous involvement being articular. The disease has a multifactorial etiology, including a major genetic predisposition, with heritability estimated at approximately 60-90% [1,2]. Approximately 60 million people worldwide suffer from this disease, accounting for a prevalence of 1.5-2% in the general population of North America and Western Europe. Psoriasis is a rare disease in children, with an estimated prevalence of around 1%, even though it is the second most frequent skin condition after atopic dermatitis in the pediatric population [1-4].

Psoriasis in both children and adults most frequently manifests as sharply demarcated erythematous papule plaques with silvery-white scales. The presence of bleeding spots if the scales are removed through scratching, known as the Auspitz sign, occurs in psoriasis. The plaques are mostly located on the extensor extremities, trunk, and scalp. This particular type is known as psoriasis vulgaris or chronic plaque psoriasis, and it occurs in approximately 75% of children with this disease [3-7]. The second most common form of psoriasis in children (15-30%) is the guttate type, more frequent in the pediatric group than in adults. It presents as small (2-6 mm diameter) widespread papules/plaques and can be misdiagnosed as lichen planus. This form can be triggered by streptococcal infection, although only 10% of children with this type are positive for anti-streptolysin O, and only 5% have a positive throat culture for *Streptococcus*. Typically, guttate psoriasis is self-limited, with resolution occurring in three to four months after the onset, but recurrence is common, and they can also evolve into psoriasis vulgaris [3-5,8,9]. Other types of psoriasis are pustular, inverse, and erythrodermic, but these types are uncommon. The diagnosis of psoriasis is mostly based on the clinical findings, but a histopathological examination (HPE) can aid the clinician. The HPE may vary with age or the evolution of the lesions, from nonspecific perivascular lymphocytic infiltration, neutrophilic aggregate, and dermal edema to epidermal hyperplasia, elongation of the rete ridges, hyperkeratosis, parakeratosis, loss of the granular cell layer, Munro's micro-abscesses, and spongiform pustules of Kogoj [1,5].

Juvenile psoriatic arthritis (JPsA) is a rare disease, affecting only 0.7-1.2% of pediatric psoriasis patients, making the diagnosis challenging. JPsA is believed to arise from a complex interaction between environmental factors, genetic predisposition, and both the adaptive and innate immune systems. This type of arthritis represents approximately 5% of juvenile idiopathic arthritis (JIA) cases. The International League of Associations for Rheumatology (ILAR) defines JPsA as either the association of arthritis and psoriasis skin lesions or by arthritis without psoriasis skin lesions but with two of the following criteria: first-degree relative diagnosed with psoriasis, dactylitis, onycholysis or nail pitting. The onset is mostly oligoarticular (<5 joints), but in the absence of treatment, 60-80% of cases extend to more than five joints (polyarticular) [10-14].

As previously stated, psoriasis is a rare disease in children (~1%), and JPsA is a rare form of presentation in psoriasis (~0.7-1.2%), making it a rare and challenging diagnosis in children with arthritis. In this paper, we present the case of a one-year-and-nine-month-old female child with sudden-onset non-traumatic arthritis accompanied by psoriasis skin lesions evident at clinical examination. The highlight of this case is the rarity of the disease, the age of onset, and the non-specific presentation.

## Case presentation

A one-year-and-nine-month-old girl was admitted to our department for a swollen left knee and recurrent fever (in the last 30 days, she had four episodes of fever, with an interval of almost one week between them). The history revealed a respiratory tract infection one month prior. After 10 days, her left knee became swollen, stiff, and warm. At the arthritis onset, three weeks prior to the admission to our hospital, she was evaluated in another facility where clinical suspicion of septic arthritis was raised, and an articular puncture was performed. However, the Gram stain and cultures of the synovial fluid were negative. During this time, she also had measles, despite being up-to-date with immunization (she received the first dose of the MMR (measles-mumps-rubella) vaccine at one year old, according to Romania's National Immunization Program).

**Figure 1:**
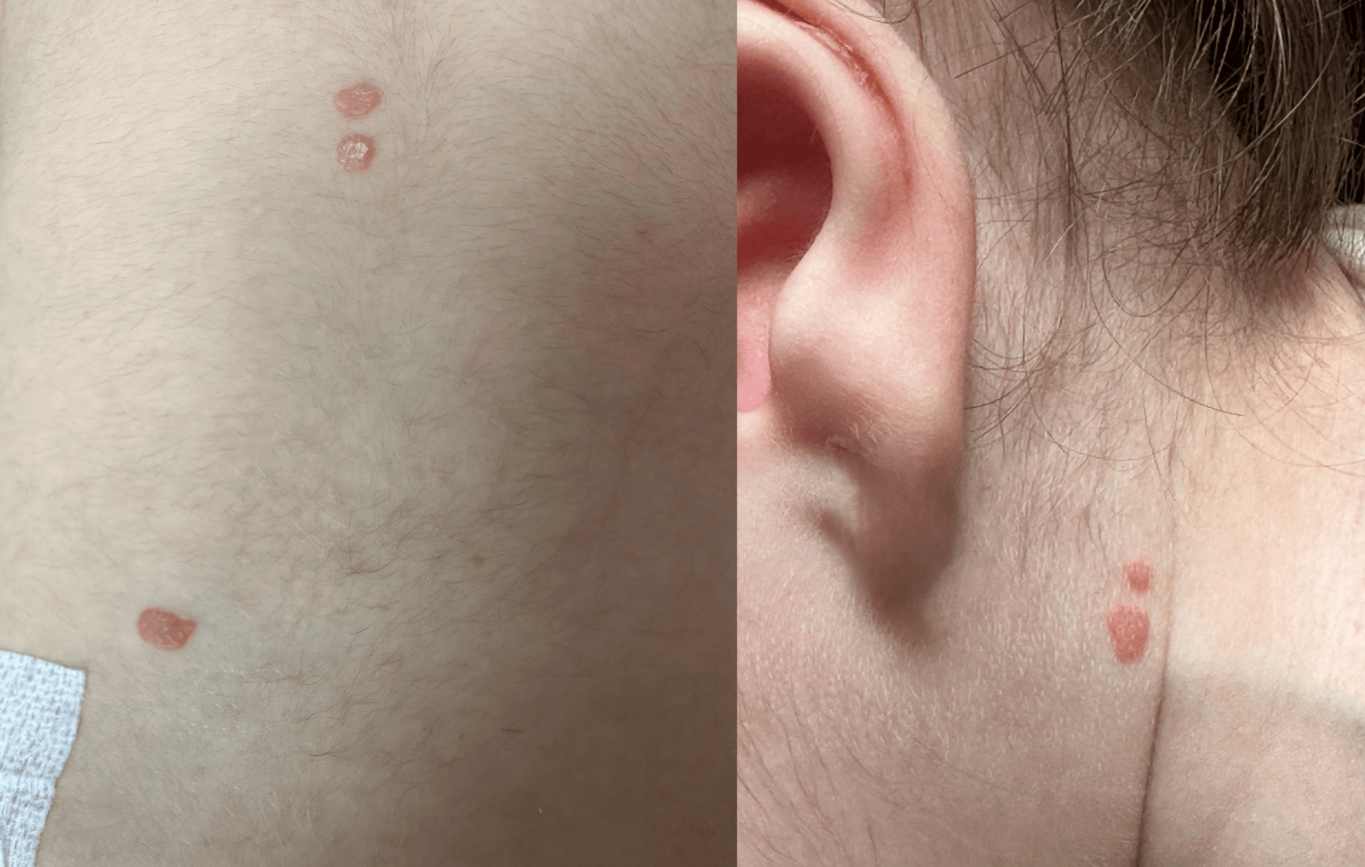
Small, red, scaly lesions located on the trunk and neck

**Figure 2:**
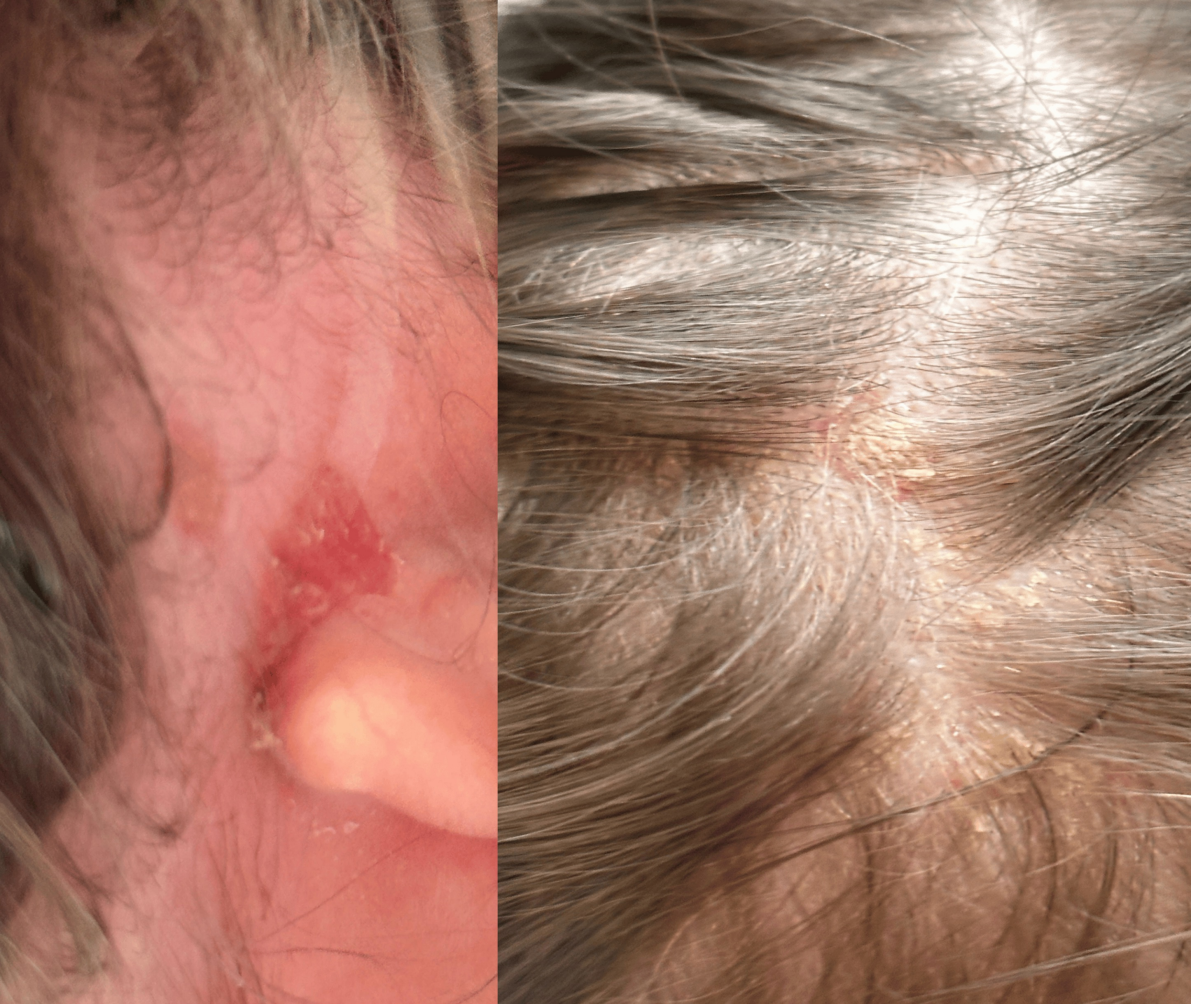
Scaly erythematous lesions located retroauricularly and on the scalp

Family history was negative for psoriasis or other autoimmune diseases. The physical examination revealed a swollen and warm left knee, multiple small, red, scaly lesions located on the trunk, scalp, and retroauricular area, and mild conjunctival hyperemia (Figures 1, 2). She was afebrile (37.1 °C) during the physical examination, with a normal heart rate (115 beats per minute) and normal respiratory rate (30 breaths per minute). With infectious arthritis excluded, we considered monoarticular early-onset juvenile idiopathic arthritis, reactive arthritis, or psoriatic arthritis as differential diagnostics.

Laboratory tests revealed mild thrombocytosis with no elevated C-reactive protein (CRP), fibrinogen, or erythrocyte sedimentation rate (ESR) levels (Table 1). Liver enzymes were within the normal range, and the screening for viral hepatitis was negative. Rubeola serology revealed positive IgG, which was a mark of the previous infection. Other infectious causes of arthritis were excluded: Lyme arthritis (negative *Borrelia burgdoferi* serology), tuberculosis (Quanti-FERON TB Gold), and post-streptococcal arthritis (negative antistreptolysin O (ASO) titer). Immunology tests revealed negative rheumatoid factor (RF), normal complement values, and normal circulating immune complexes. Anti-citrullinated protein antibodies (ACPAs), anti-*Saccharomyces cerevisiae* IgA and IgG (ASCA), antinuclear antibodies (ANA), anti-myeloperoxidase antibodies (anti-MPO) were also normal. The thyroid hormone profile was normal (thyroid-stimulating hormone and thyroxine), and anti-thyroperoxidase antibodies were negative, showing no evidence of autoimmune thyroiditis.

**Table 1:** Laboratory findings

Parameters	Results	Normal Values
White blood cells	10.37	4–12×10^9^/L
Neutrophils	5.22	2–8×10^9^/L
Lymphocytes	4.37	0.8–7×10^9^/L
Monocytes	0.69	0.12–1.20×10^9^/L
Eosinophils	0.06	0.02–0.80×10^9^/L
Basophils	0.03	0–0.10×10^9^/L
Hemoglobin	12.10	12–16 g/dL
Platelets	441	150–400×10^9^/L
Erythrocyte sedimentation rate	12	7–12 mmol/hour
C-reactive protein	0.31	0–0.5 mg/dL
Fibrinogen	286.70	140 400 mg/dL

**Figure 3:**
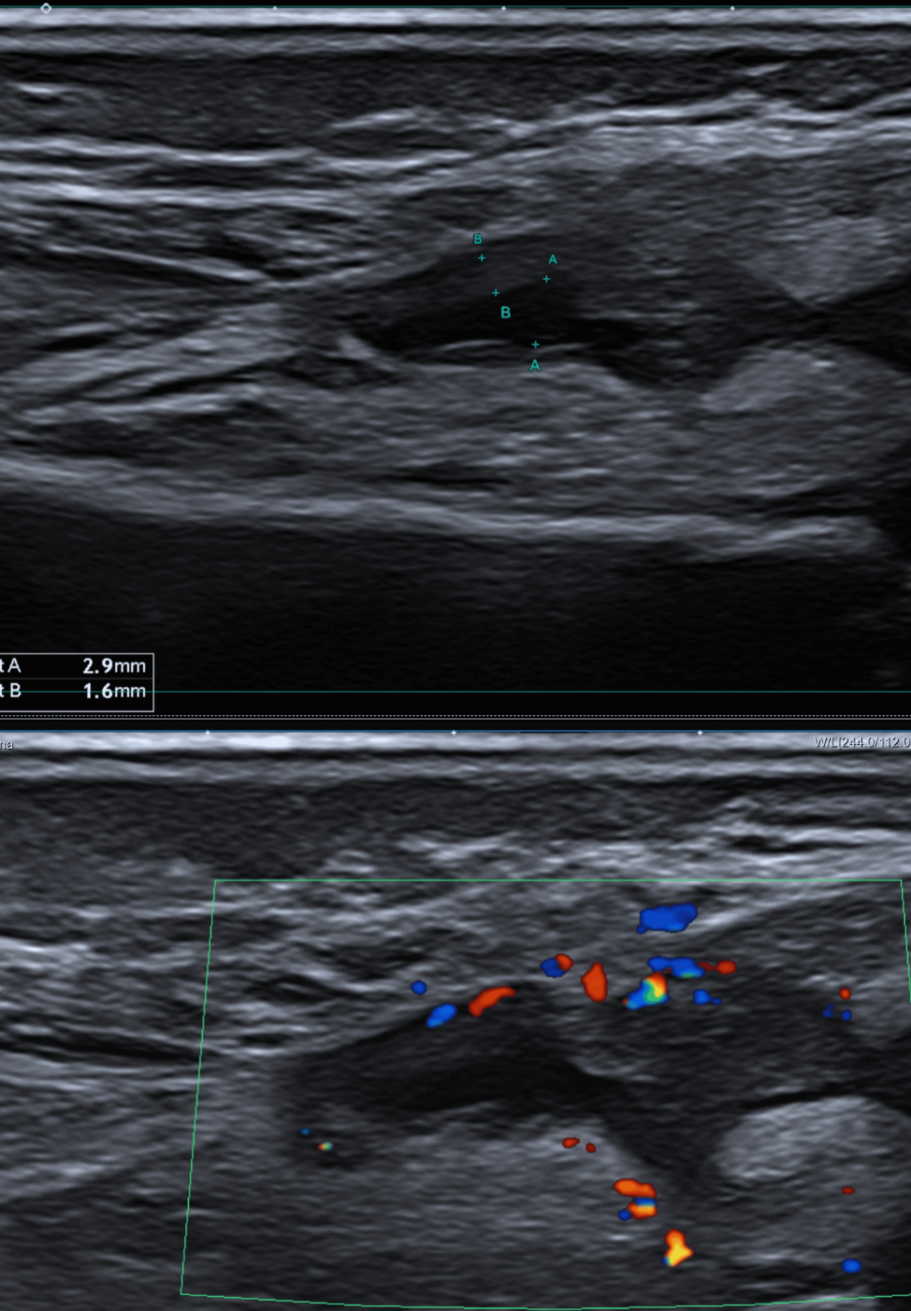
Ultrasound of the left knee

The ultrasound of the swollen knee showed a thickened synovial membrane (4 mm), with hyperemia, suggestive of synovitis, and a small quantity of synovial fluid (3-4 mm) in the suprapatellar recess. The peripatellar fat tissue was hyperechogenic (Figure 3).

The ophthalmological exam revealed mild conjunctivitis and no uveitis.

**Figure 4:**
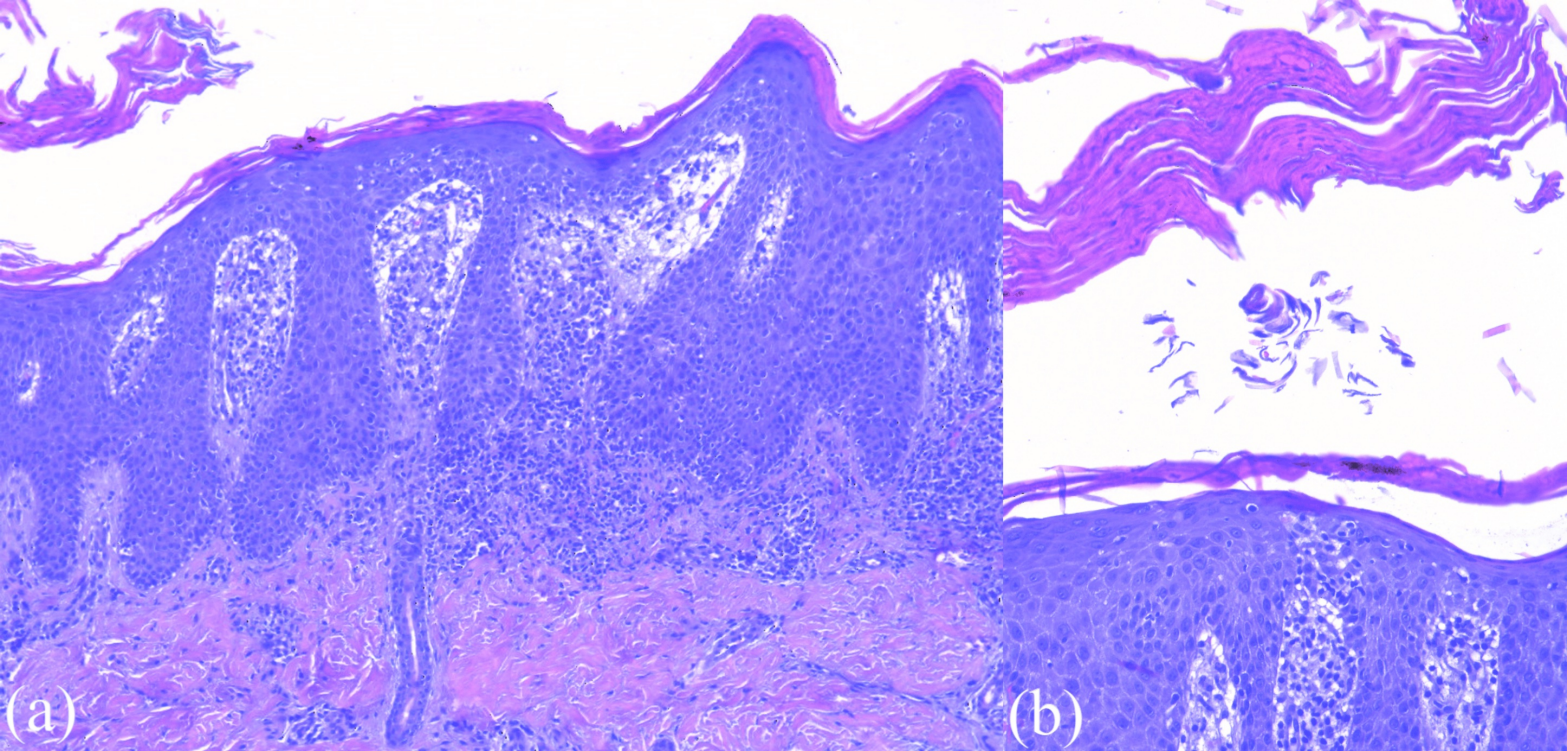
Histological examination (1) (a) Hematoxylin and eosin (H&E) stain (50×) shows the epidermis with regular acanthosis with elongated rete ridges (psoriasiform); (b) H&E stain (200×) shows thinning of the suprapapillary plates and parakeratosis in the stratum corneum in the epidermis.

**Figure 5:**
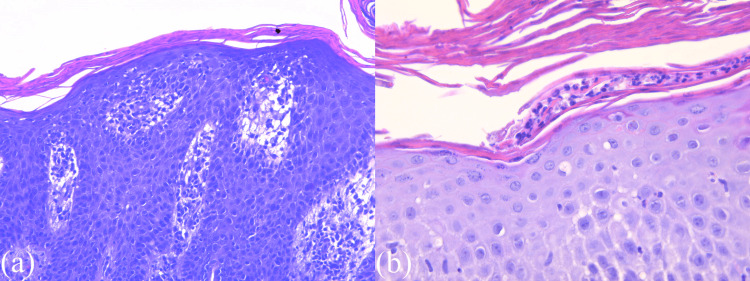
Histological examination (2) (a) Hematoxylin and eosin (H&E) stain (200×) shows a perivascular, predominantly lymphocytic infiltrate in papillary dermis with few intraepidermal lymphocytes; (b) H&E stain (400×) shows an area of parakeratosis in the stratum corneum with mounds of neutrophils (Munro's micro-abscesses).

Dermatological examination described sharply demarcated erythematous papules covered with white-yellowish scales, punctiform vessels, and a positive Auspitz sign, suggestive of psoriasis. The clinical diagnosis was guttate psoriasis. A skin biopsy was performed, and the histopathological examination showed epidermal psoriasiform acanthosis with parakeratosis and superficial inflammatory cells (polymorphonuclear neutrophils) (Figure 4). The examination of the papillary dermis showed perivascular mixed inflammatory infiltrate with lympho-monocytes and neutrophils, with marked hyperemia. The epidermis appeared thin and spongy, exhibiting lymphocyte exocytosis (Figure 5).

With macroscopic and microscopic findings suggestive of psoriasis and monoarthritis, we established the diagnosis of psoriatic arthritis. The patient was further referred to a specialty clinic for treatment, where she received weekly injections of methotrexate (MTX), followed by oral administration of folic acid for six months, then she started oral therapy with MTX. She also received oral therapy with ibuprofen and omeprazole for two months, when she was administered an intra-articular injection of triamcinolone. The patient had complete remission of arthritis and psoriasis skin lesions after three months of treatment.

## Discussion

The described case meets the ILAR criteria for JPsA, presenting with psoriasis skin lesions and arthritis [13]. The age of onset was one year and eight months, and the literature describes a bimodal peak in early childhood, around two years old, and in a time frame of 10-12 years. A study published in 2017 by Zisman et al. that included 361 JPsA patients enrolled in the Childhood Arthritis and Rheumatology Research Alliance (CARRA) registry reported that the average age of symptom onset was 8.34 ± 4.57 years, and 22.7% of them were defined as early-onset (<4 years old) [15]. The average age at onset of those in the early-onset group was 3.12 ± 2.08 years. Another study published by Butbul Aviel et al., which included 115 JPsA patients, found a similar average age of onset, 8.0 ± 4.4 years. This case is part of the early-onset minority group and had even an earlier onset than the average reported [11,12,15].

There was no family history of psoriasis in this case. The percentage of relevant family history in JPsA described in the literature varied from 31.3% to 59%. Although a family history of psoriasis is typically associated with an early onset of the disease, this is not the case in the present case [12,15,16].

The type of psoriasis in this case was the guttate one. This type is described in the literature as the second most common type after plaque psoriasis, ranging from approximately 14% to 30% of pediatric psoriasis cases [4,17-19]. Guttate psoriasis is stated in the literature to be associated with a recent streptococcal infection or an upper respiratory tract infection. In this case, the throat culture and the ASO titer were negative, but the patients reported an upper respiratory tract infection 10 days before the onset of the disease [3,5,8].

The psoriasis lesions in this case were located on the scalp, trunk, and retroauricular areas. A cross-sectional observational clinical study conducted by Al Hamdi, which included 416 children with psoriasis, reported that the most frequent presentation is scalp psoriasis (16.8%). Usually, when the scalp is affected, lesions can also be found behind the auricles [20,21].

The JPsA in this respective case was one with an oligoarticular onset. Butbul Aviel et al. reported that the majority of JPsA patients from their cohort, 65 (55%) of 115, had an oligoarticular onset. However, Zisman et al. reported that in a cohort of 361 JPsA patients, the oligoarticular group was the second most frequent (44.7%). The joint involved in this case was the left knee. In the previously mentioned study by Butbul Aviel et al., the knee joint was the most frequent articulation involved at onset (66.9%). Another study published in 2006 by Stoll et al. described similar results, with knee joint involvement in 66% of cases out of 70 patients with JPsA [12,15,22].

Although uveitis is the most common extra-articular manifestation of JPsA, children with JPsA can also develop inflammatory conjunctivitis. Early-onset JPsA is a risk factor for ocular involvement. Nail changes are less frequent in children than in adults but can be identified in approximately 40% of cases. Nail alterations include pitting, trachyonychia, onycholysis, oil spots, and subungual hyperkeratosis. Zisman et al. reported in their 361 cohort other manifestations, such as nail pitting and dactylitis, in almost one-third of cases (37.5% and 29.7%, respectively) and uveitis in 11.2%. No other manifestations aside from the psoriasis lesions, conjunctivitis, and arthritis were present in our case, but uveitis can develop several years after the onset of articular and skin lesions, so ophthalmologic follow-up is necessary [15,23-27].

The CRP in the described case had a normal range value. Many patients with PsA do not have elevated values of CRP despite the presence of inflammation. Sokolova et al. found that only 33% of 15 adult PsA presented an increased value of the CRP. Butbul Aviel et al. described a minor elevation mean value of 0.6±1 mg/dL in their JPsA patients [12,28-30].

The RF was negative, a finding consistent with the literature where the rate of positive RF is small (3-4.7%). ANA results were also negative, with literature indicating that almost half of the cases (~46%) exhibit positive ANA, highlighting an association between the age of onset and the positivity of these antibodies. In Zisman et al.'s study, the positivity rate of ANA was higher in the early-onset group than that in the late-onset group (61.8% vs. 41.4%, p=0.003), while Butbul Aviel et al. also found an association between ANA positivity and younger age (R^2^=0.07; p=0.0027, OR: 0.862, CI: 0.78-0.953) [12,15,22].

## Conclusions

JPsA is a rare disease characterized by numerous characteristics related to the history, age of onset, and clinical and laboratory data, making it a challenging diagnosis, especially in young children. Although rare, JPsA may appear in children younger than two years old. Arthritis may precede the psoriasis skin lesion, necessitating time for the diagnosis of JPsA.
